# Should Global Burden of Disease Estimates Include Depression as a Risk Factor for Coronary Heart Disease?

**DOI:** 10.1186/1741-7015-9-47

**Published:** 2011-05-03

**Authors:** Fiona J Charlson, Nicholas JC Stapelberg, Amanda J Baxter, Harvey A Whiteford

**Affiliations:** 1 Queensland Centre for Mental Health Research, Cnr Ellerton Drive and Wolston Park Rd, Wacol, Qld 4074, Australia; 2 School of Population Health, University of Queensland, Herston Road, Herston, Qld, 4006, Australia; 3 School of Psychology and Griffith Health Institute, Griffith University, Parklands Drive, Southport, Qld 4215, Australia

## Abstract

The 2010 Global Burden of Disease Study estimates the premature mortality and disability of all major diseases and injuries. In addition it aims to quantify the risk that diseases and other factors play in the aetiology of disease and injuries. Mental disorders and coronary heart disease are both significant public health issues due to their high prevalence and considerable contribution to global disease burden. For the first time the Global Burden of Disease Study will aim to assess mental disorders as risk factors for coronary heart disease. We show here that current evidence satisfies established criteria for considering depression as an independent risk factor in development of coronary heart disease. A dose response relationship appears to exist and plausible biological pathways have been proposed. However, a number of challenges exist when conducting a rigorous assessment of the literature including heterogeneity issues, definition and measurement of depression and coronary heart disease, publication bias and residual confounding. Therefore, despite some limitations in the available data, it is now appropriate to consider major depression as a risk factor for coronary heart disease in the new Global Burden of Disease Study.

## Background

Mental disorders and coronary heart disease are both significant public health issues due to their high prevalence and considerable contribution to global disease burden. The 2001 Global Burden of Disease (GBD) Study ranked unipolar depressive disorders as the third leading cause of disease burden, rising to first place for high- and middle-income countries [1]. In spite of this ranking, the burden of depression may still be underestimated because of inadequate appreciation of the links between depression and other health conditions, such as ischemic heart disease [2].

Ischemic heart disease (IHD) is a major cause of disease burden, ranked fourth globally and second in high- and middle-income countries [1]. A number of cohort studies in recent years have contributed to the growing body of evidence for links between mental disorders and cardiovascular disease. The largest body of work in this area has been done on the association between major depressive disorder (MDD) and coronary heart disease (CHD), with results implying the existence of a robust association [3-7].

Establishing whether depression is an independent risk factor for CHD could have a significant impact on resource allocation and treatment for both depressive and some cardiovascular diseases. Previous GBD studies have included illicit drug use and alcohol use as risk factors for outcomes as diverse as IHD, stroke, hypertensive disease, depression, and intentional and unintentional injury [8]. Mental disorders other than substance abuse have not been incorporated in GBD studies as risk factors to-date.

The 2010 GBD Study currently underway http://www.globalburden.org/ views risk factors in the context of several criteria: evidence of a causal effect should be established, but the risk should be not too broadly defined (for example diet or environment as a whole) or be too narrow (for example every single fruit and vegetable or every toxicant in tobacco smoke). There should be a relatively specific definition of risk factor exposure and risk factors should be potentially modifiable. Sufficient data on risk factor exposure and risk factor disease relationships should exist. Finally, protective as well as hazardous factors should be considered.

In their 2007 paper, Prince *et al *outline implications for health policy and resourcing based on the under-recognized burden of mental disorders as a risk factor for other disease [2]. The relationship between depression and CHD has been well studied and it is reasonable to consider it for inclusion as a risk factor for CHD in a GBD study. There have been studies on the relationship between depression and other disease entities falling under the umbrella of vascular disease, other than CHD. These studies, for example on the relationship between depression and stroke (CVA), fall beyond the scope of this review. The objective of this paper is to review the available evidence on the role of depression as an independent risk factor in CHD and recommend whether this evidence base is sufficiently robust to include depression as an independent risk factor for CHD in the new GBD Study.

## Discussion

### Is the Direct Association Between Depression and CHD Plausible?

#### Biological Mechanisms

Depression has various direct and indirect biological effects which may explain its link with cardiovascular disease. Current evidence suggest that depression may act through an autonomically-mediated increase in myocardial workload (and thus ischemia), or in ventricular irritability; diminished beat-to-beat heart rate variability; or increased atherogenesis, through relatively direct effects on platelet function or hypercortisolemia-mediated effects on other cardiovascular risk factors such as blood pressure or lipid levels [9,10]. Vascular endothelial dysfunction of the coronary arteries has also been observed in a number of cohorts of depressed patients [11].

Depression-related dysregulation of the sympathoadrenal system and HPA axis has been observed to cause an increase in circulating catecholamines and serum cortisol resulting in surges in heart rate and blood pressure, which increases the risk of atherosclerotic plaque rupture and acute coronary thrombosis [11]. High levels of catecholamines also increase the irritability of the heart muscle which can lead to ventricular arrhythmias and modify the function of circulating platelets [9,11]. Increased cortisol levels lead to inflammation, excessive clotting, metabolic syndrome, induction of hypercholesterolemia and hypertryglyceridemia, and injury of vascular endothelial cells [2,9]. Effects on serotonin metabolism have also been shown to lead to additional alteration of cardiac function, increased platelet activity, and vasoconstriction [2]. Changes in inflammatory immune response, such as raised inflammatory markers, may also predict the development of cardiovascular disease [2,11].

#### Behavioral Mechanisms

A number of behavioral pathways have been identified in the link between mental disorders and cardiovascular disease. Patients who suffer from a mental disorder are less likely to adopt a healthy lifestyle or to reduce cardiovascular risk factors. Physical activity and diet control are less frequently undertaken and smoking and alcohol consumption rates are often higher [2,11-13]. In addition, obesity has been strongly associated with both cardiovascular disease and mental disorders such as depression, bipolar disorder and panic disorder [2]. Adherence to treatment is often less in depressed patients compared to non-depressed patients [11]. Depressed patients are known to access social services less frequently and insufficient social support has been strongly linked to the development of cardiovascular disease [11].

#### Temporal Relationship

Establishing the temporal relationship of depression antecedent to CHD is a challenge. Both depression and early stages of cardiovascular disease can go undiagnosed for a substantial period of time. In addition, the relationship between depression and CHD appears to be bi-directional. Increased prevalence of depression post-cardiac event and its negative prognostic effect on cardiac patients has been clearly demonstrated [14,15]. Proof of a temporal relationship can be provided by only including prospective studies in which patients with previous CHD are excluded from the study, or at the very least excluded from the analysis. However, Rugulies and colleagues [5] tested this hypothesis by pooling effect sizes for all relevant studies and then for studies which control for sub-threshold CHD by intensive medical examinations at baseline. They report a small reduction in the effect size (from 1.77 to 1.51) but it remains statistically significant.

It can further be argued that if sub-threshold CHD were present at baseline, relative risk (RR) of outcome would be higher within a short period of time and tail off with longer follow-up. The literature does not support this. Data from the John Hopkins Precursors Study showed that risk of myocardial infarction (MI) after 40 years of follow-up did not differ from risk after only 10 years (RR of 2.12 and 2.1, respectively) [16]. Implications of these findings are that possible sub-threshold CHD at baseline does not significantly affect the risk of incident CHD in depressed samples.

#### Evidence for Association between Depression and CHD

Several reviews of the association between depression and incident CHD have been published. Specifically four meta-analyses have been published, reporting pooled relative risk for depression and incident CHD between 1.57 and 2.69 [3,5-7]. A further four qualitative reviews were identified, all reporting a high degree of evidence for the role of depression in the development of CHD [13-15,17]. A number of authors conclude that the risk of depression on incident CHD may be equal to that of smoking [6,7,17].

Three other qualitative reviews [9,11,12] document increased cardiovascular morbidity and mortality in patients with depressive symptoms or major depression. A strong suggestion of a dose-response relationship between depression and CHD was identified [5,7,11,14]. Depression meeting diagnostic criteria was associated with a higher risk of CHD compared to depressive symptoms [5,7]. Existence of a dose-response relationship between exposure and outcome supports likelihood of causation [15].

#### Association is stronger with MI than more broadly defined CHD

A comprehensive meta-analysis based on 28 studies reported a pooled relative risk of 1.60 (95%CI 1.34-1.92) for MI compared to 1.48 (95%CI 1.29-1.69) and 1.63 (1.26-2.12) for CHD and CVD, respectively [7]. It was only when the outcome was narrowed to MI alone that heterogeneity between studies was eliminated. This suggests that classification of outcome (MI), as opposed to other factors was an important source of heterogeneity between the studies. An important limitation of this review is that not all included studies controlled for CHD at baseline. Yet the implication of this finding is that the evidence for depression as an independent risk factor is more consistent for incident MI than for CHD.

#### Heterogeneity Issues

The literature on depression and cardiovascular disease displays large heterogeneity. Lack of consistency in methodology makes the task of comparing studies and pooling data for meta-analysis difficult and needs to be carefully considered in any systematic review. For example, it has been shown that the prognostic impact of initial depression increases over time, with higher odds ratios for mortality post diagnosis of depression in CHD in studies with longer follow-up periods [18].

#### Defining and Measuring Depression

For the purposes of the GBD, depressive disorders are required to meet the Diagnostic and Statistical Manual of Mental Disorders (DSM) [19] diagnostic criteria for major depressive disorder (F32-F33) or dysthymic disorder (F34.1), or the WHO International Classification of Diseases (ICD)[20] criteria for depressive disorders including dysthymia (F32.0-F32.2, F32.8, F33.0-F33.2, F33.8, F33.9, F34.1).

However, identification and measurement of depression is complex and the refinement of adequate, quantitative measures is ongoing. Measures of depression used in published studies range from self-report of mood to strict adherence to diagnostic criteria, with a myriad of other surveys and questionnaires somewhere between and varying on the spectra of reliability and validity [11,21].

There exists variation in the cut-off values of 'diagnostic' scoring within studies which use the same measurement instrument [22-25]. The ability to compare findings from various studies employing differing measurement methods is therefore compromised. Case identification according to DSM or ICD criteria is the ideal. Where a study falls short of this, for example in large cohort studies, a diagnosis by a clinician or the use of a measurement tool which has been shown to acceptably represent DSM or ICD criteria is considered sufficient for the case ascertainment of depression. Additional complication comes about by variations in the period of assessment (for example, depression experienced during the past-week versus past 12-months).

#### Defining and Measuring Coronary Heart Disease

CHD is considered to encompass ischemic heart disease, myocardial infarction and angina, included cases of ICD-9 codes 410-414 and 429, or ICD-10 codes 120-125. The WHO criteria for myocardial infarction (MI) are also accepted. While the term Coronary Heart Disease was recommended by Tunstall-Pedoe (2001) [26], many studies dealing with the relationship between depression and disease of the coronary vessels use alternative nomenclature, such as Coronary Artery Disease (CAD) or Ischemic Heart Disease (IHD). All essentially refer to the disease process of atherosclerosis of the coronary arteries leading to myocardial infarction and angina [27].

MI provides a very well defined clinical marker, both in terms of a clearly definable 'hard' endpoint for cardiovascular disease [12], as well as a marker in time, allowing temporal correlation between MI and depressed mood.

Although case-definitions for CHD are clear in theory, signs of CHD can be very difficult to detect and subclinical in nature. Hence ruling out CHD at baseline and proving an unbiased etiological relationship is challenging [28]. This is particularly true for angina and it is for this reason that angina was not accepted as a proxy for CHD.

### Has the Evidence Base Been Affected by Publication Bias?

The possibility of accurately measuring depression as a risk factor for CHD appears to be hampered by significant publication bias. The apparent over-representation of positive studies showing an increased risk could in fact be hiding truer lesser risk of CHD in depressed subjects [3].

### Is There Residual Uncontrolled Confounding?

Although the obvious confounding variables should be controlled for as a requirement for inclusion in a quantitative review, residual uncontrolled confounding cannot be ruled out due to the complex nature of the depression and CHD relationship. For example, a depressive state may be associated with other CVD risks such as smoking and other chronic medical conditions.

Reviews of the literature reporting associations between psychosocial factors and CHD found strength of evidence for depression as a causative factor to be strong compared to anxiety where the effect was less consistent [2,15]. A role for anxiety in the development of CHD is biologically plausible. Anxiety, as with depression, affects the autonomic regulation of the heart via the hypothalamic-pituitary-adrenocorticol (HPA) axis [10,29]. Chronic over-activation of these systems due to anxiety would help to explain the increased frequency of cardiovascular risk factors in patients with anxiety disorders [10]. Reduced heart rate variability in those with anxiety disorders could also involve impaired vagal control [12]. In the hypertensive state a chronic anxiety disorder may trigger increased levels of epinephrine and norephinephrine which could also increase the heart rate, total peripheral resistance and even increase the circulating fatty acids [10]. There is significant evidence which links anxiety disorders to sudden cardiac death [30,31]. However, anxiety has not been associated with myocardial infarction in these studies [12,31].

While some reviews have found an association between anxiety and CHD [32], they included fewer of the primary studies with negative findings than the review by Kuper *et al *[15,33]. We have concluded there is neither strong nor consistent evidence for a causal association between anxiety disorders and CHD [33].

However, depression and anxiety are strongly co-morbid [34,35]. Hence we need to consider anxiety as a confounder in the relationship between depression and CHD. Given the conclusion above that anxiety disorders (alone) do not have a proven etiological relationship with CHD we examined the evidence for the relationship between major depressive disorder and CHD controlling for other mental disorders. In one study the odds ratio (OR) for incident MI was only slightly reduced once phobia, panic disorder and alcohol use/dependence were controlled for (4.16 95%CI 1.49-11.62 compared to 4.54 95%CI 1.65-12.44) [36]. We therefore concluded that there is insufficient evidence for co-morbid anxiety substantially mediating the relationship between depression and CHD.

A number of studies have reported an association between use of anti-depressant medication and fatal and nonfatal CHD [37,38]. This raises the issue as to whether anti-depressant medication, rather than depression, is a risk factor in development of CHD. A limitation of many earlier papers considering the role of antidepressants and CHD [37,38] is that few controlled for the presence of depression or other mental disorders. More recent studies examining depression and CHD have controlled for the use of medications. Retrospective analysis of a primary care sample with IHD found that use, dose and duration of tricyclic anti-depressant consumption were not statistically significant in the development of ischemic heart disease when diagnosis and duration of depression were included in the regression model [39]. In a prospective follow-up of the Baltimore Epidemiologic Catchment Area reported use of psychotropic medication had a marginal effect on the RR of incident MI in a depressed sample (RR 4.16 compared to 4.14) [36].

## Summary

Should major depression be considered an independant etiological risk factor in the development of CHD for global burden of disease estimates?

There is strong evidence that major depression is an independent risk factor for CHD. The evidence for this has emerged recently and this field is still being actively explored. Studies of the relationship between major depression and CHD frequently have significant differences in their methodology and there are potential biases which may have affected study outcomes.

Several potential confounders such as smoking, weight, physical activity, blood pressure and cholesterol [40] have led to controversy over of the effect size measured in some studies. However, when confounders are accounted for MDD remains strongly associated with at least a doubling in risk of cardiac events over 1 to 2 years after an MI [41].

There is sufficient evidence to recommend that Burden of Disease estimates should include MDD as a risk factor for CHD, and this evidence is strongest for CHD resulting in MI. There are now several large-scale studies from different countries and settings which have reported consistent findings. We conclude the association between MDD and CHD is very unlikely to be due to chance or bias [11]. We agree with Goldston and Baillie [11] that the existence of several plausible pathophysiological mechanisms for the relationship, the strength and consistency of the association and the finding that it is dose-related supports the conclusion that depression is an independent etiological risk factor in CHD.

What is needed is further research to clarify the nature of this relationship and specifically to further quantify it. Issues surrounding methodological heterogeneity could be addressed by adopting standard methodologies. Some such standards have already been published [42,43]. It is vital however that further awareness is raised about major depression as a risk factor for CHD. We believe the inclusion of major depression as a risk factor for CHD in the 2010 GBD study will raise awareness about the relationship and encourage the collection of international data for more accurate ascertainment of the risk. Given that major depression has emerged as a risk factor which confers an only slightly less attributable risk than that of lifetime smoking and a comparable attributable risk to that of both hypertension and obesity [4], such action is warranted.

In a broader context, there is also an urgent need for further well-designed and targeted research examining other mental disorders as independent risk factors for cardiovascular disease. Specific areas identified where future research is needed:

• More and better quality studies with attention to improved methodological consistencies,

• Examination of risk by country and region - more research in low- and middle-income countries,

• Clearer understanding of behavioral and biological pathways and interrelationship of different factors,

• Further validation of and consistencies between measurement instruments,

• Differentiating between the effects of depression, anxiety and substance use disorders in the development of CHD,

• The impact that pharmacological and/or non-pharmacological treatment of depression has on CHD outcomes.

## Abbreviations

CHD: Coronary heart disease; DSM: Diagnostic and Statistical Manual of Mental Disorders; GBD: Global Burden of Disease; HPA: Hypothalamic-pituitary-adrenal; ICD: International Classification of Disease; IHD: Ischaemic heart disease; MDD: Major depressive disorder; MI: Myocardial infarction; OR: Odds ratio; RR: Relative risk; WHO: World Health Organisation

## Competing interests

Harvey Whiteford is Chair of the Mental Disorders Expert Group for the 2010 Global Burden of Disease Study. Amanda Baxter is the Project Manager for the Mental Disorders Expert Group for the 2010 Global Burden of Disease Study.

## Authors' contributions

FC Made a substantial contribution to conception and design of the paper, and acquisition and interpretation of data; played a principal role in drafting the article and revising it critically for important intellectual content; and performed final critical editing and gave approval of the version to be published. CS made a substantial contribution to conception and design of the paper, and acquisition and interpretation of data; played an important role in drafting the article and revising it critically for important intellectual content; and performed final critical edit and gave approval of the version to be published. AB made a substantial contribution to conception and design of paper and assisted in the acquisition of data; revised it critically for important intellectual content, and performed a final critical edit; and gave approval of the version to be published. HW made a substantial contribution to conception and design of paper and assisted in the acquisition of data; revised it critically for important intellectual content, and performed a final critical edit; and gave approval of the version to be published.

## Pre-publication history

The pre-publication history for this paper can be accessed here:

http://www.biomedcentral.com/1741-7015/9/47/prepub
